# Cellular Mechanisms of Myocardial Depression in Porcine Septic Shock

**DOI:** 10.3389/fphys.2018.00726

**Published:** 2018-06-12

**Authors:** Dagmar Jarkovska, Michaela Markova, Jan Horak, Lukas Nalos, Jan Benes, Mahmoud Al-Obeidallah, Zdenek Tuma, Jitka Sviglerova, Jitka Kuncova, Martin Matejovic, Milan Stengl

**Affiliations:** ^1^Department of Physiology, Faculty of Medicine in Pilsen, Charles University, Pilsen, Czechia; ^2^Biomedical Center, Faculty of Medicine in Pilsen, Charles University, Pilsen, Czechia; ^3^Department of Internal Medicine I, Faculty of Medicine in Pilsen, Charles University, Pilsen, Czechia; ^4^Department of Anesthesiology and Intensive Care Medicine, Faculty of Medicine in Pilsen, Charles University, Pilsen, Czechia

**Keywords:** sepsis, pig, myocardial depression, calcium, mitochondria

## Abstract

The complex pathogenesis of sepsis and septic shock involves myocardial depression, the pathophysiology of which, however, remains unclear. In this study, cellular mechanisms of myocardial depression were addressed in a clinically relevant, large animal (porcine) model of sepsis and septic shock. Sepsis was induced by fecal peritonitis in eight anesthetized, mechanically ventilated, and instrumented pigs of both sexes and continued for 24 h. In eight control pigs, an identical experiment but without sepsis induction was performed. *In vitro* analysis of cardiac function included measurements of action potentials and contractions in the right ventricle trabeculae, measurements of sarcomeric contractions, calcium transients and calcium current in isolated cardiac myocytes, and analysis of mitochondrial respiration by ultrasensitive oxygraphy. Increased values of modified sequential organ failure assessment score and serum lactate levels documented the development of sepsis/septic shock, accompanied by hyperdynamic circulation with high heart rate, increased cardiac output, peripheral vasodilation, and decreased stroke volume. In septic trabeculae, action potential duration was shortened and contraction force reduced. In septic cardiac myocytes, sarcomeric contractions, calcium transients, and L-type calcium current were all suppressed. Similar relaxation trajectory of the intracellular calcium-cell length phase-plane diagram indicated unchanged calcium responsiveness of myofilaments. Mitochondrial respiration was diminished through inhibition of Complex II and Complex IV. Defective calcium handling with reduced calcium current and transients, together with inhibition of mitochondrial respiration, appears to represent the dominant cellular mechanisms of myocardial depression in porcine septic shock.

## Introduction

Sepsis represents a well-recognized worldwide health problem. Based on the meta-analysis of studies from developed high-income countries, global annual estimates were 31.5 million sepsis and 19.4 million severe sepsis cases, with potentially 5.3 million deaths, in the hospital setting (Fleischmann et al., 2016). Sepsis plays a prominent role in hospital mortality: in two large complementary inpatient cohorts (total of 157,518 deaths/7,038,449 admissions) sepsis was found to contribute to 1 in every 2 to 3 deaths (Liu et al., 2014).

Sepsis is defined as life-threatening organ dysfunction caused by a dysregulated host response to infection (Singer et al., 2016). A common manifestation of sepsis/septic shock is myocardial depression with reversible biventricular dilatation and depressed ejection fraction (Antonucci et al., 2014). The precise mechanistic link between infection, sepsis, and myocardial depression remains unclear, although a number of possible pathways have been suggested (Merx and Weber, 2007; Sato and Nasu, 2015). An early theory of global myocardial ischemia due to inadequate coronary blood flow in sepsis was disproved by findings of high coronary blood flow and diminished coronary artery–coronary sinus oxygen difference in septic patients (Cunnion et al., 1986). Nowadays, there is a general consensus on the mechanism of a circulating depressant substance, as originally demonstrated by Parrillo et al. (1985). The exact nature of the circulating depressant substance, however, has not been sufficiently clarified yet. The list of possible candidates includes various cytokines, endotoxins, prostanoids, and nitric oxide (Merx and Weber, 2007; Sato and Nasu, 2015).

Similarly, the downstream cellular pathophysiology of myocardial depression is still obscure, although in experimental models, a number of contributing mechanisms have been reported. The cardiac cellular mechanisms include a reduction of L-type calcium current (I_CaL_) (Lew et al., 1996; Zhong et al., 1997; Stengl et al., 2010), altered calcium transients (Ren et al., 2002), increased calcium leakage from the sarcoplasmic reticulum (Zhu et al., 2005), impaired sarcolemmal diastolic calcium extrusion pathways (Wagner et al., 2015), oxidation and subsequent activation of calcium and calmodulin-dependent protein kinase with phosphorylation of the ryanodine receptor (Sepúlveda et al., 2017), altered phosphorylation and calcium sensitivity of cardiac myofibrillar proteins (Wu et al., 2001), and/or mitochondrial dysfunction (Levy et al., 2004; Watts et al., 2004).

Most data on the intrinsic cellular mechanisms of myocardial depression, however, were obtained in small animal (rodent) experimental models with limited clinical relevance and translatory potential (Poli-de-Figueiredo et al., 2008; Dyson and Singer, 2009). To overcome this limitation, cellular mechanisms of myocardial depression were examined in a clinically relevant porcine model of peritonitis-induced progressive septic shock, which, in contrast to rodent hypodynamic endotoxic shock, closely mimics human sepsis (hyperdynamic circulation with low systemic vascular resistance and multiple organ dysfunction). The myocardial functions were examined on several levels of biological complexity, from the *in vivo* experiment similar to the clinical scenario down to experiments in isolated cells and organelles, with special emphasis on calcium homeostasis and mitochondrial function.

## Materials and Methods

Animal handling was in accordance with the European Directive for the Protection of Vertebrate Animals Used for Experimental and Other Scientific Purposes (86/609/EU). The experiments were approved by the Committee for Experiments on Animals of the Charles University Faculty of Medicine in Pilsen and by the Ministry of Education, Youth and Sports of the Czech Republic (Protocol No. MSMT-24725/2014-05). All experiments were performed in the animal research laboratory at the Faculty of Medicine in Pilsen. Sixteen domestic pigs of both sexes and of similar weight (43.9 ± 5.8 kg) were used for experiments. Sepsis was induced by fecal peritonitis in eight pigs (seven boars, one sow) while control sham experiments (analogous procedure but without sepsis induction) were performed in another eight pigs (four boars, four sows).

### Anesthesia and Instrumentation

Anesthesia and instrumentation protocols were similar to those previously described (Jarkovska et al., 2016). Anesthesia was induced with intramuscular (IM) tiletamine (2.2 mg/kg), zolazepam (2.2 mg/kg), and xylazine (2.2 mg/kg), together with intravenous (IV) propofol 2% (1–2 mg/kg) and maintained with continuous IV propofol (1–4 mg/kg/h) and fentanyl (5–10 μg/kg/h). Animals were mechanically ventilated (FiO_2_ 0.3, PEEP 8 cm H_2_O, tidal volume 10 ml/kg, respiratory rate adjusted to maintain end/tidal pCO_2_ between 4 and 5 kPa), and muscle paralysis was achieved with IV rocuronium (4 mg for induction, 0.2–0.4 mg/kg/h for maintenance). Ringerfundin solution (B. Braun Melsungen AG, Melsungen, Germany) was infused as maintenance fluid (7 ml/kg/h) and normoglycemia (arterial blood glucose level 4.5–7 mmol/L) was maintained using 10% glucose infusion (1–4 ml/kg/h).

All pigs were instrumented with a femoral artery catheter, triple lumen central venous catheter, and pulmonary artery catheter. Silicone drains directed into the anatomical spaces of Morison and Douglas were used for fecal inoculation.

### Experimental Protocol

Experimental protocols were identical to those previously described (Jarkovska et al., 2016). Peritonitis was induced by inoculating 1 g/kg of autologous feces (cultivated for 10 h in 100 ml isotonic saline at 37°C) into the abdominal cavity. In addition to continuous crystalloid infusion, fluid boluses (10 ml/kg of Ringerfundin) were administered to maintain cardiac output and mean arterial pressure (MAP) in a goal-directed fashion. Continuous IV norepinephrine was administered if MAP fell below 65 mmHg despite fluid administration and titrated to maintain MAP above 70 mmHg. In total, the experiments lasted 34 h (4 h for surgical instrumentation, 6 h of recovery, and 24 h after induction of peritonitis). At the end of the experiment, the animals were euthanized by anesthetic overdose and excision of the heart.

### Measurements

Systemic and pulmonary hemodynamics were measured and electrocardiography (lead II) was performed as described previously (Stengl et al., 2010; Jarkovska et al., 2016). The modified sequential organ failure assessment (SOFA) score was determined according to the Third International Consensus Definitions for Sepsis and Septic Shock (Singer et al., 2016) and modified by exclusion of the Glasgow Coma Scale-based neurologic component.

Action potentials and isometric contractions in the right ventricle trabeculae were recorded as described previously (Stengl et al., 2008, 2010, 2013). Action potentials were recorded with high-resistance (>20 MΩ) glass microelectrodes filled with 3 M KCl at various stimulation frequencies (3, 2, 1, 0.5 Hz), and simultaneously, isometric contractions were recorded using an isometric force transducer (F30, Hugo Sachs, March-Hugstetten, Germany). All action potential [APD90, action potential duration at the 90% level of repolarization, action potential amplitude (APA), resting membrane potential (RMP)] and contraction (contraction force, time from resting tension to the peak of contraction, time to 90% relaxation) parameters were measured in 5 beats and averaged, and the mean values were used for further analyses and comparisons.

Cardiac myocytes were isolated from the left ventricle by enzymatic dissociation (collagenase A from Sigma-Aldrich, St. Louis, MO, United States) as previously reported (Stengl et al., 2010). I_CaL_ was measured using the whole-cell configuration of the patch-clamp technique at 36°C (Stengl et al., 2010).

Sarcomeric contractions and calcium transients of isolated cardiac myocytes were measured with Ionoptix HyperSwitch Myocyte Calcium and Contractility System (IonOptix LLC, Westwood, CA, United States), with the Sarclen sarcomere length acquisition module. Cells were loaded with Fura-2 (Molecular Probes, Invitrogen, Waltham, MA, United States). For stock solution Fura-2-am powder was dissolved in dimethyl sulfoxide (DMSO; Sigma-Aldrich, St. Louis, MO, United States) to reach a final concentration of 1 mmol/L. Cells were incubated for 20 min in normal calcium Tyrode solution with 2 μmol/L Fura-2-am and then repeatedly washed with normal calcium Tyrode solution. Measurements were performed in normal Tyrode solution at 37 ± 0.5°C. Cells were stimulated with a field stimulator (MyoPacer Field Stimulator, IonOptix LLC, Westwood, CA, United States) at frequencies of 3, 2, 1, 0.5 Hz. For offline analysis of sarcomeric contractions and calcium transients, the IonWizard 6.5 software (IonOptix LLC, Westwood, CA, United States) was used.

Cardiac mitochondrial function was assessed using high-resolution respirometry (oxygraph Oroboros; Oroboros Instruments, Innsbruck, Austria). Samples of left ventricular myocardium (1.5–2.0 mg) were permeabilized by saponin in BIOPS solution (Cantó and Garcia-Roves, 2015). The fibers were then washed with respiration medium containing catalase and placed into oxygraph chambers with MiR06 medium equilibrated with air. Mitochondrial oxygen consumption was measured at 37°C after raising oxygen concentration to 400–500 μmol/L by titration of H_2_O_2_ (200 mmol/L). In the titration protocol, different substrates and inhibitors of the mitochondrial respiratory system were sequentially added into the chambers to determine various respiratory states; non-phosphorylating LEAK state (L, oxygen consumption needed for electron transport compensating for proton leak across the inner mitochondrial membrane, induced by the addition of substrates providing electrons to Complex I – malate, 2 mmol/L, glutamate, 10 mmol/L, and pyruvate, 5 mmol/L), OXPHOS I (active phosphorylating respiration induced by 5 mmol/L ADP), OXPHOS Ic (the degree of cell membrane permeabilization verified with cytochrome c 10 μmol/L), OXPHOS I+II (mitochondrial respiration increased by succinate, Complex II substrate, 10 mmol/L), OXPHOS II (reflecting activity of Complex II, induced by inhibition of Complex I by rotenone, 0.5 μmol/L), ROX (residual oxygen consumption after complex III inhibition by antimycin A, 2.5 μmol/L), and Complex IV activity (respirometric assay for cytochrome c oxidase activity by simultaneous injection of *N,N,N′,N′*-tetramethyl-*p*-phenylenediamine dihydrochloride, TMPD, 0.5 mmol/L, and ascorbate, 2 mmol/L).

The oxygen consumption was analyzed online by DatLab software (Oroboros Instruments, Innsbruck, Austria) as the negative time derivative of oxygen concentration in the chamber, expressed in pmol O_2_/(s⋅mg tissue wet weight), and corrected to ROX.

Citrate synthase activity serving as a marker of mitochondrial content was measured in all samples taken from the oxygraph chambers. The assay medium was mixed with homogenized chamber content and citrate synthase activity was measured spectrophotometrically at 412 nm and 30°C, and expressed in IU per g tissue weight (Kuznetsov et al., 2002).

### Solutions and Drugs

The composition of the Tyrode solution was as follows (in mmol/L): NaCl 137, KCl 4.5, MgCl_2_ 1, CaCl_2_ 2, glucose 10, HEPES 5; pH was adjusted to 7.4 with NaOH. The patch-clamp pipette solution contained: cesium glutamate 125, tetraethylammonium chloride 25, MgCl_2_ 1, Na_2_ATP 5, EGTA 1, HEPES 5; pH adjusted to 7.2 with CsOH. BIOPS solution was composed of CaK_2_EGTA 2.77, K_2_EGTA 7.23, Na_2_ATP 5.77, MgCl_2_⋅6H_2_O 6.56, taurine 20, Na_2_ Phosphocreatine 15, imidazole 20, dithiothreitol 0.5, and MES hydrate 50, with pH adjusted to 7.1. (Pesta and Gnaiger, 2012). MiR06 respiration medium contained EGTA 0.5, MgCl_2_⋅6H_2_O 3, K-lactobionate 60, taurine 20, KH_2_PO_4_ 10, HEPES 20, sucrose 110, fatty acid free bovine serum albumin 1 g/L, and catalase 280 U/mL at pH 7.0 (Gnaiger et al., 2000). The composition of the assay medium for determination of citrate synthase activity was 5,5-dithio-*bis*-(2-nitrobenzoic) acid 0.1, oxaloacetate 0.5, acetyl coenzyme A 0.31, triethanolamine hydrochloride 5, Tris-HCl 100, and 5 μmo/L EDTA, Triton-X 0.25%, pH adjusted to 8.1). All chemicals were from Sigma-Aldrich (St. Louis, MO, United States).

### Statistical Analysis

Results are presented as means ± SD. After testing for normality of distribution (Shapiro–Wilk test), statistical comparisons were made with the two-way mixed-design ANOVA (one repeated-measures factor for analysis of the progression of parameter in time and one between-groups factor for comparison between control and septic groups, *in vivo* data) followed by *post hoc* Tukey test or by unpaired *t*-test (control vs. septic group comparison of *in vitro* data). The analysis was performed using the software Origin 2017 (OriginLab, Corp., Northampton, MA, United States) or STATISTICA Cz 8 (StatSoft, Inc., Prague, Czechia). Values of *p* < 0.05 were considered significant.

## Results

**FIGURE 1:**
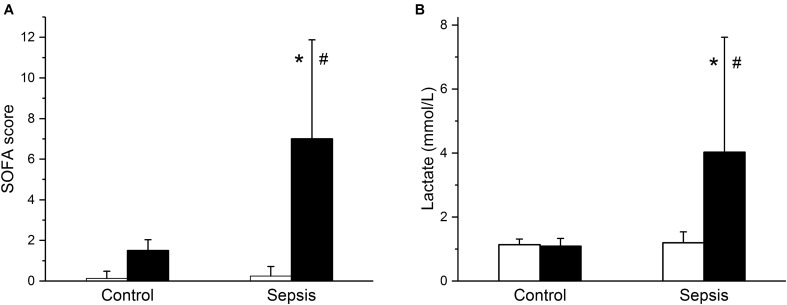
Modified sequential organ failure assessment (SOFA) score and serum lactate. **(A)** Modified SOFA score in control (*n* = 8) and septic (*n* = 8) animals. Empty columns, start of experiment; filled columns, end of experiment. **(B)** Serum lactate levels in control (*n* = 8) and septic (*n* = 8) animals. Empty columns, start of experiment; filled columns, end of experiment. ^∗^ Significantly different from start of the experiment, *p* < 0.05; # significantly different from control, *p* < 0.05.

**FIGURE 2:**
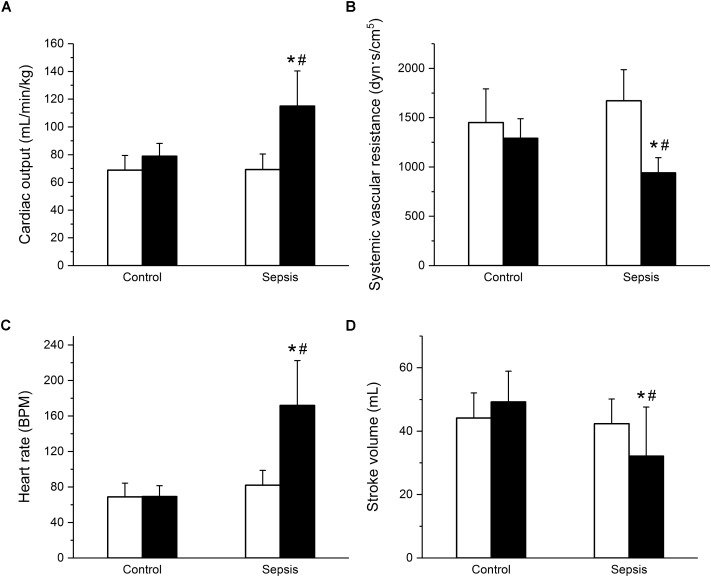
Hyperdynamic circulation in sepsis. **(A)** Cardiac output in control (*n* = 8) and septic (*n* = 8) animals. Empty columns, start of experiment; filled columns, end of experiment. **(B)** Systemic vascular resistance in control (*n* = 8) and septic (*n* = 8) animals. Empty columns, start of experiment; filled columns, end of experiment. **(C)** Heart rate in control (*n* = 8) and septic (*n* = 8) animals. Empty columns, start of experiment; filled columns, end of experiment. **(D)** Stroke volume in control (*n* = 8) and septic (*n* = 8) animals. Empty columns, start of experiment; filled columns, end of experiment. ^∗^ Significantly different from start of the experiment, *p* < 0.05; # significantly different from control, *p* < 0.05.

In the septic group, development of sepsis and septic shock with organ dysfunction was manifested by a significant increase of the total modified SOFA score (**Figure 1A**) and of serum lactate levels (**Figure 1B**). Out of eight animals in this group, septic shock requiring vasopressors and with lactate levels over 2 mmol/L developed in six animals; in two animals, vasopressor administration was not required, but the criteria for sepsis was fulfilled based on their SOFA scores. Concurrently, the septic animals developed hyperdynamic circulation with increased cardiac output and peripheral vasodilation (**Figures 2A,B**). The increase in cardiac output was predominantly due to elevated heart rate (**Figure 2C**), as the stroke volume was reduced (**Figure 2D**). The control animals did not show any signs of systemic inflammatory reaction, their SOFA scores remained normal (**Figure 1A**), their lactate levels remained low throughout the run of the experiment, and none of them needed vasopressor support. Their global hemodynamic parameters remained unchanged compared to the baseline (**Figures 2A–D**).

**FIGURE 3:**
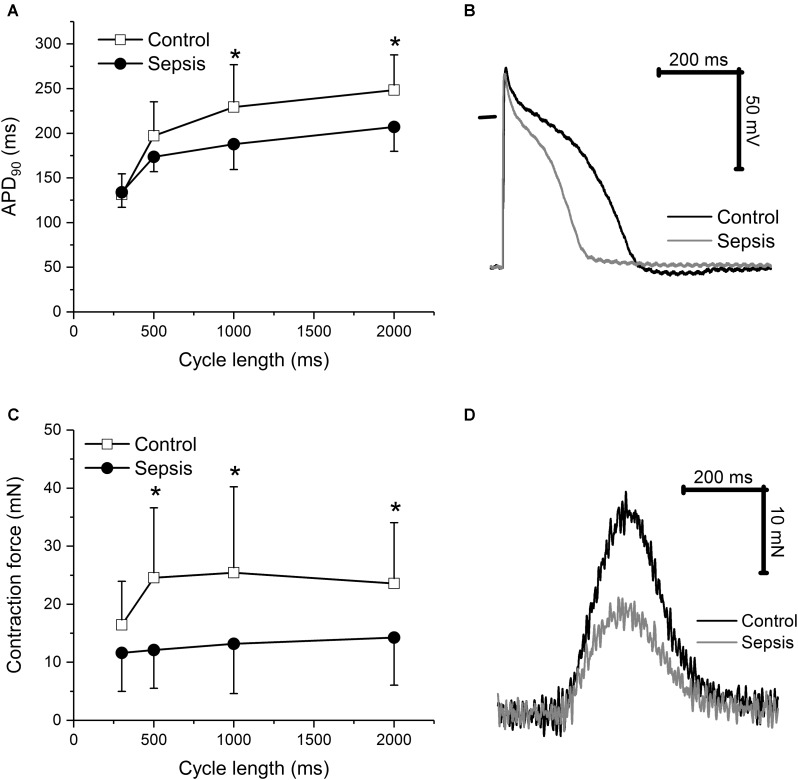
Action potential and contraction in cardiac trabeculae. **(A)** Action potential duration at 90% repolarization in cardiac trabeculae from control (*n* = 8) and septic (*n* = 8) animals. Empty squares, control; filled circles, sepsis. **(B)** Representative action potentials of cardiac trabeculae from control and septic animals. Stimulation frequency of 1 Hz. Black line, control; gray line, sepsis. **(C)** Contraction force in cardiac trabeculae from control (*n* = 8) and septic (*n* = 8) animals. Empty squares, control; filled circles, sepsis. **(D)** Representative contractions of cardiac trabeculae from control and septic animals. Stimulation frequency of 1 Hz. Black line, control; gray line, sepsis. ^∗^ Significantly different from control, *p* < 0.05.

In cardiac trabeculae, sepsis induced a shortening of action potential duration (**Figures 3A,B**) at lower stimulation rates (1, 0.5 Hz) and reduction of contraction force (**Figures 3C,D**). Values of APA and RMP were similar in control and septic preparations (e.g., at 1 Hz APA of 101 ± 5 mV in control vs. of 100 ± 12 mV in sepsis; RMP of -71 ± 7 mV in control vs. -70 ± 7 mV in sepsis). Kinetics of trabecular contraction (time to peak, TTP; time to 90% relaxation, R90) were not affected by sepsis (e.g., at 1 Hz TTP of 184 ± 53 ms in control vs. 171 ± 57 ms in sepsis; R90 of 243 ± 80 ms in control vs. 240 ± 61 ms in sepsis).

**FIGURE 4:**
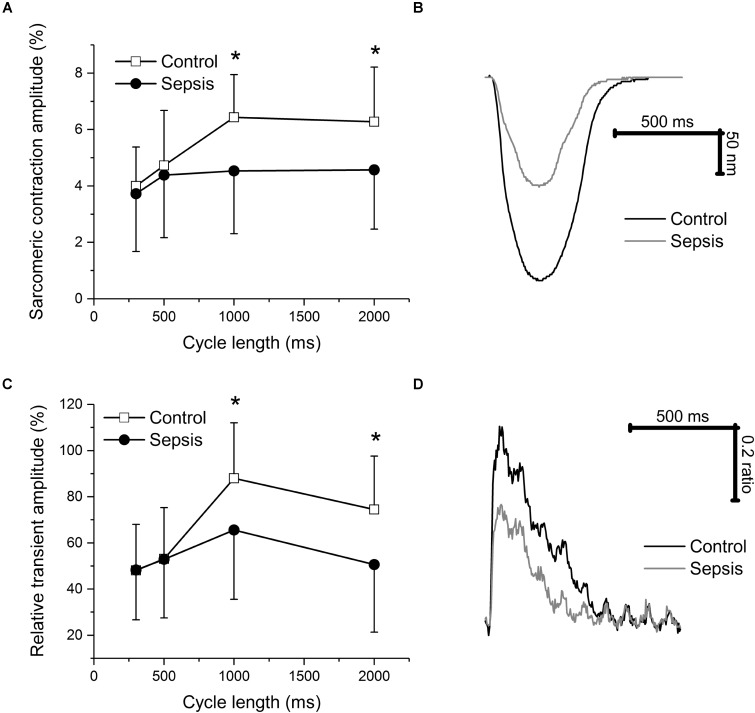
Sarcomeric contraction and calcium transients in cardiac myocytes. **(A)** Relative sarcomeric contraction amplitudes in cardiac myocytes from control and septic animals. Empty squares, control (*n* = 14); filled circles, sepsis (*n* = 21). **(B)** Representative sarcomeric contractions of cardiac myocytes from control and septic animals. Stimulation frequency of 1 Hz. Black line, control; gray line, sepsis. **(C)** Relative calcium transient amplitudes in cardiac myocytes from control and septic animals. Empty squares, control (*n* = 14); filled circles, sepsis (*n* = 21). **(D)** Representative calcium transients of cardiac myocytes from control and septic animals. Stimulation frequency of 1 Hz. Black line, control; gray line, sepsis. ^∗^ Significantly different from control, *p* < 0.05.

**FIGURE 5:**
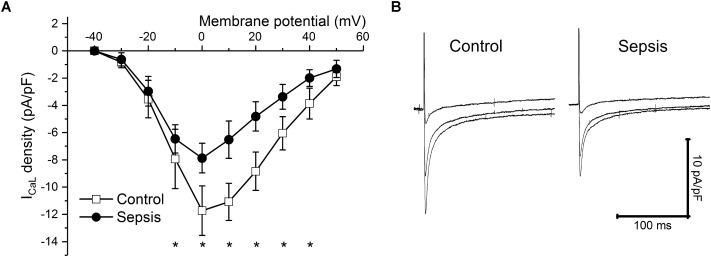
I_CaL_ in sepsis. **(A)** I_CaL_ density at various membrane potentials (–40 to +50 mV) in control (empty squares, *n* = 10) and septic (filled circles, *n* = 10) cardiac myocytes. ^∗^ Significantly different from control, *p* < 0.05. **(B)** Representative I_CaL_ traces at membrane potentials of 0, +20, and +40 mV of cardiac myocytes from control and septic animals. Left panel, control; right panel, sepsis.

In isolated myocytes, sarcomeric contractions (**Figures 4A,B**) and calcium transients (**Figures 4C,D**) were reduced in septic cells at lower stimulation rates (1, 0.5 Hz). The resting intracellular calcium concentrations were not affected by sepsis at any stimulation frequency (e.g., at 1 Hz ratio of 0.588 ± 0.084 in control vs. 0.623 ± 0.115 in septic myocytes). Kinetic parameters of both sarcomeric contractions and calcium transients (time to 50% peak, TP50; time to 50% relaxation, TR50) were not affected by sepsis (e.g., for sarcomeric contractions at 1 Hz, TP50 of 78 ± 27 ms in control vs. 84 ± 29 ms in septic myocytes; TR50 of 365 ± 117 ms in control vs. 391 ± 104 ms in septic myocytes; for calcium transients at 1 Hz, TP50 of 12 ± 3 ms in control vs. 12 ± 3 ms in septic myocytes; TR50 of 316 ± 90 ms in control vs. 322 ± 110 ms in septic myocytes). I_CaL_ was decreased in septic cardiac myocytes at membrane potentials between -20 and +40 mV (**Figures 5A,B**).

**FIGURE 6:**
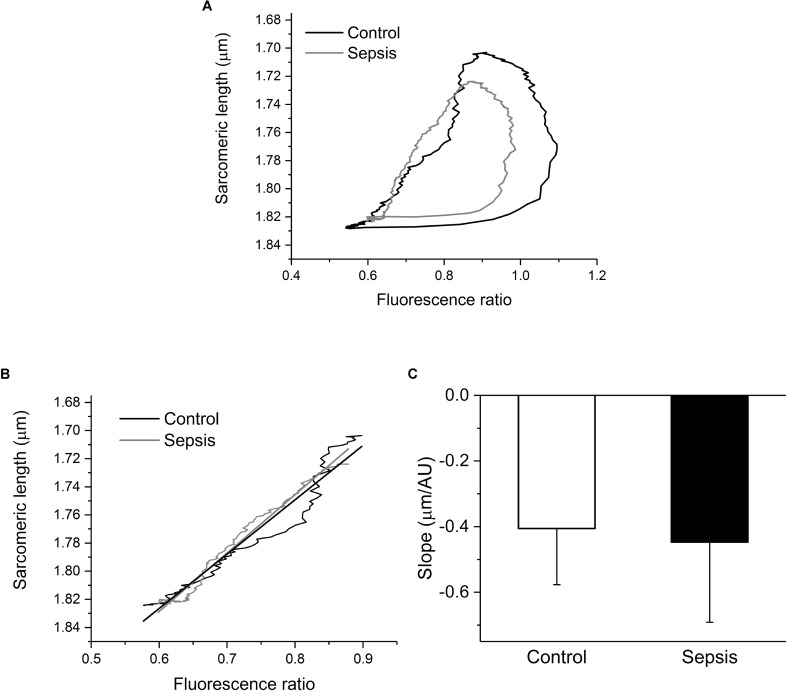
Phase-plane diagrams of sarcomeric length-intracellular Ca^2+^ relationship. **(A)** Mean phase-plane trajectories of sarcomeric length-fluorescence ratio relationship in control (black line, *n* = 7) and septic (gray line, *n* = 8) myocytes. **(B)** Amplified relaxation phase of the mean trajectories with linear fits. Black lines, control; gray lines, sepsis. **(C)** Slopes of linear fits of relaxation portions of phase-plane trajectories. Empty column, control; filled column, sepsis.

Since sarcomeric contractions and calcium transients were recorded simultaneously in each cardiac myocyte, phase-plane trajectories of sarcomeric length-intracellular Ca^2+^ relationship were constructed (**Figure 6A**) and analyzed. The sarcomeric length-intracellular Ca^2+^ trajectory during the relaxation phase of the twitch contraction was similar in control and septic myocytes (**Figure 6B**). Linear fitting of these relaxation phase trajectories revealed similar slopes (**Figure 6C**) for control and septic myocytes.

**FIGURE 7:**
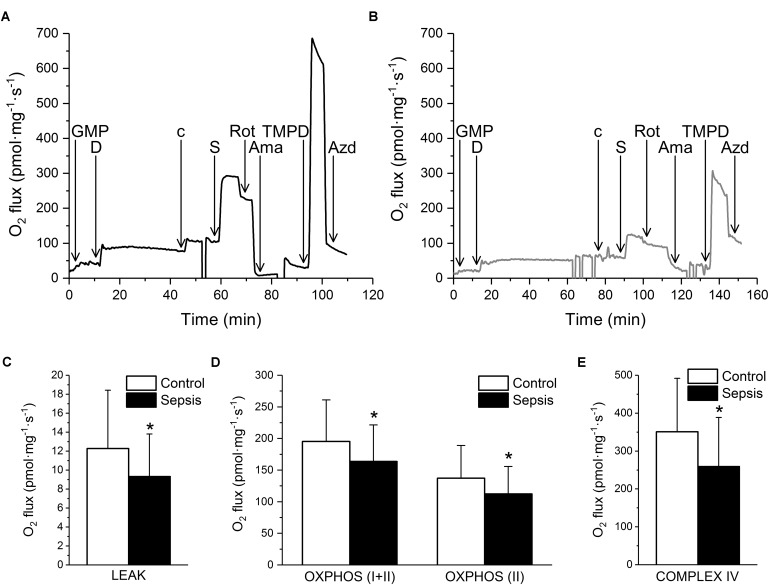
Mitochondrial respiration in sepsis. **(A)** Representative trace of oxygen consumption during substrate-inhibitor titration protocol in control myocardium. **(B)** Representative trace of oxygen fluxes during substrate-inhibitor titration protocol in septic myocardium. GMP – glutamate, malate, pyruvate, D – ADP, c – cytochrome c, S – succinate, Rot – rotenone, Ama – antimycin A, TMPD – *N,N,N*′*,N*′-tetramethyl-*p*-phenylenediamine dihydrochloride, AZD – sodium azide. **(C)** Oxygen consumption on LEAK state in control (empty column, *n* = 33 samples/5 pigs) and septic (filled column, *n* = 44 samples/6 pigs) myocardium. **(D)** Oxygen consumption in OXPHOS (I+II) and OXPHOS (II) states in control (empty columns, *n* = 33 samples/5 pigs) and septic (filled columns, *n* = 44 samples/6 pigs) myocardium. **(E)** Complex IV oxygen consumption in control (empty column, *n* = 33 samples/5 pigs) and septic (filled column, *n* = 44 samples/6 pigs) myocardium. ^∗^ Significantly different from control, *p* < 0.05.

Mitochondrial respiration was suppressed in septic hearts (**Figure 7**). Oxygen consumption in the LEAK state was decreased (**Figure 7C**). Mitochondrial respiration in the presence of ADP and Complex I and II substrates (OXPHOS I+II) was reduced by sepsis, and this reduction was mainly due to inhibition of Complex II (OXPHOS II), while Complex I activity was not influenced significantly (**Figure 7D**). Cytochrome c oxidase (Complex IV) activity was also decreased in sepsis (**Figure 7E**).

Citrate synthase activity was not affected by sepsis, reaching 64.2 ± 12 and 62.1 ± 11 IU/g in control and septic samples, respectively.

## Discussion

In our clinically relevant porcine model of peritonitis-induced sepsis/septic shock, we have managed to induce the typical hyperdynamic circulation pattern, with high heart rate but reduced stroke volume and low systemic vascular resistance. On the cellular level, this was associated with shortened action potential duration, decreased contraction force and calcium transient amplitude, and reduced I_CaL_. Analysis of phase-plane diagrams of sarcomeric length versus calcium concentration (fluorescence ratio) indicated no change in myofilament calcium sensitivity. Mitochondrial respiration was suppressed in septic hearts, predominantly due to an inhibition of Complexes II and IV.

To the best of our knowledge, this is the first complex analysis of cellular and subcellular mechanisms of septic myocardial depression in a clinically relevant large animal (porcine) experimental model. In earlier studies, the cellular effects of sepsis on excitation–contraction coupling in the myocardium were only studied in small animal (rodent) models. Consistent with our porcine data, septic peritonitis rat model cardiac myocytes exhibited a depression of both peak shortening and calcium transients (Ren et al., 2002). Similarly, decreased myocyte shortenings and peak systolic calcium levels, together with slowed-down kinetics of calcium transients, were described in isolated cardiac ventricular cardiomyocytes of rats with sepsis induced by cecal ligation and puncture (Zhu et al., 2005). In mice with sepsis due to colon ascendens stent peritonitis, cardiac myocytes showed reduced cell shortening, calcium transient amplitude, and sarcoplasmic reticulum calcium content, which was associated with a significant increase in oxidation-dependent calcium and calmodulin-dependent protein kinase II activity (Sepúlveda et al., 2017). In general, the data obtained in rodent models of sepsis/endotoxemia indicate an important role of intracellular calcium homeostasis in septic myocardial depression.

In this study of porcine septic shock, the reduction of cardiac contractile force (documented in multicellular preparations of cardiac trabeculae, as well as in isolated cardiac myocytes) was associated with decreased amplitude of calcium transient, reduced I_CaL_, and shortened action potential duration. I_CaL_ represents the main entry pathway of calcium into the cardiac myocyte and is crucial for both triggering calcium release from the sarcoplasmic reticulum and replenishing intracellular calcium stores during the plateau phase of the cardiac action potential (Eisner et al., 2017). Reduction of I_CaL_, together with the shortening of action potential duration, results in a substantial suppression of the calcium influx (Stengl et al., 2010). Similar kinetics of the rising phase of calcium transients in control and septic myocytes suggest that triggering of the sarcoplasmic reticulum calcium release was not significantly affected by the reduction of I_CaL_, leaving the diminished I_CaL_ calcium influx for replenishing intracellular calcium stores and/or direct stimulation of contractile proteins as the most likely mechanism.

Another possible contributor to septic myocardial depression might be the altered functional properties of myofibrillar proteins. In rats with cecal ligation and puncture sepsis, the phosphorylation of both troponin I and of C protein was increased during the early phase but decreased during the late phase of sepsis (Wu et al., 2001). The decreases in the phosphorylation of troponin I and C protein during late sepsis coincided with the declines in the activities of myofibrillar ATPase and the calcium sensitivity of myofilaments. Similarly, in rabbit non-lethal endotoxemia, a phosphorylation-dependent decrease in myofibrillar calcium sensitivity was documented (Tavernier et al., 2001). In contrast to these findings, septic plasma from a canine model of Escherichia coli sepsis failed to decrease isometric tension in the skinned trabecular preparations with chemically disrupted sarcolemmal, sarcoplasmic reticulum, and mitochondrial membranes, which ruled out a direct inhibition of the contractile apparatus by septic plasma (Gu et al., 1998). In line with the canine sepsis data, the phase-plane analysis of sarcomeric length-intracellular Ca^2+^ relationship revealed no significant change of myofilament responsiveness in porcine septic shock. The intracellular Ca^2+^-cell length trajectory during the relaxation phase of the twitch contraction in single cardiac myocytes defines a quasi-equilibrium of cytosolic calcium, myofilament Ca^2+^ binding, mechanical force, and cell length (Spurgeon et al., 1992). Since the position and the slope of the relaxation phase trajectories were virtually identical in control and septic myocytes, the calcium responsiveness of myofilaments was probably not affected in our experimental setting of porcine sepsis.

The growing consensus that mitochondrial dysfunction contributes to the pathogenesis of sepsis and development of septic cardiomyopathy is mainly based on studies performed in small laboratory animals (Cimolai et al., 2015). Decreased respiratory rates and/or reduced activities of respiratory mitochondrial complexes were reported in septic rabbits (Gellerich et al., 2002), rats (Vanasco et al., 2012), and mice (Piquereau et al., 2013). In majority of studies, inhibition of Complex I was documented, while inhibition of other Complexes was less consistent. Only two studies published so far addressed the myocardial mitochondrial respiratory dysfunction in porcine models of peritonitis-induced sepsis: one of them reported reduced activity of Complex I determined spectrophotometrically at 30°C and expressed per citrate synthase activity (Li et al., 2007); the other documented no difference in myocardial mitochondrial oxygen consumption between control and septic animals treated with antibiotics for at least 48 h (Corrêa et al., 2012). In our current study, suppression of mitochondrial respiration related to Complexes I and II (OXPHOS I+II) could be attributed mainly to inhibition of Complex II. Oxygen consumption by artificially stimulated Complex IV was also reduced, while activity of Complex I was not significantly influenced. Expression of oxygen consumption per citrate synthase activity did not affect the pattern of changes. Decreased LEAK state with reduced mitochondrial respiration expressed per mg tissue wet weight would suggest reduced mitochondrial content and/or swelling of the tissue due to fluid resuscitation; however, unchanged citrate synthase activity argues against these options. The experimental discrepancies are probably related to species differences, as well as variable experimental protocols (models of sepsis, duration of sepsis, severity of insult). Is there a direct link between abnormal calcium handling and mitochondrial dysfunction? It is well-known that cardiac sarcoplasmic reticulum and mitochondria closely interact, forming a mitochondrial calcium microdomain (Kohlhaas and Maack, 2013). The sarcoplasmic reticulum Ca^2+^-ATPase preferentially consumes mitochondrial ATP for active transport of cytosolic calcium back to the sarcoplasmic reticulum (Kaasik et al., 2001). On the other hand, calcium released from the sarcoplasmic reticulum enters the mitochondria, even on a beat-to-beat basis (Andrienko et al., 2009), and regulates mitochondrial enzymes of the tricarboxylic acid cycle, the proteins of the electron transport chain, and the ATP synthase (Williams et al., 2015), thus matching energy supply to demand. Accordingly, in our study, decreased contraction and calcium release were accompanied by decreased mitochondrial respiration, but the question remains as to what the primary event is. In our opinion, the facts that the kinetics of calcium transients decline and the myofilament calcium responsiveness were not affected by sepsis argue against the primary role of the mitochondria and insufficient energy supply, and rather indicate defective calcium transport (reduced I_CaL_ with consequent alterations of sarcoplasmic reticulum calcium release) as the primary event of myocardial depression.

## Conclusion

Defective calcium handling with reduced calcium current and transients, together with inhibition of mitochondrial respiration, appear to represent the dominant cellular mechanisms of myocardial depression in porcine septic shock. Consequently, these molecular mechanisms may help to identify potential therapeutic targets for preventing and/or reversing sepsis-induced myocardial dysfunction. In this line of thinking, calcium channel openers represent an obvious option. Bay K 8644, a dihydropyridine calcium channel agonist, was shown to enhance I_CaL_ in cardiac myocytes from endotoxemic rats (Abi-Gerges et al., 1999). *In vivo*, BAY K 8644 elevated blood pressure in endotoxin-shocked rats (Ives et al., 1986) as well as in endotoxemic dogs (Preiser et al., 1991). On the other hand, in cardiac myocytes from endotoxemic guinea pigs BAY K 8644 did not reverse the endotoxin-induced reduction in peak I_CaL_, cell contraction, and systolic intracellular calcium concentration (Zhong et al., 1997). Mitochondrial respiration is another promising target for potential therapeutic interventions. In endotoxemic rat models, mitochondria-targeted antioxidants were demonstrated to reduce inflammatory responses, mitochondrial damage and organ dysfunction including cardiac depression (Supinski et al., 2009; Lowes et al., 2013).

## Study Limitations

The modified SOFA score was determined according to human sepsis criteria (Singer et al., 2016). Despite generally similar physiology and sepsis progression in this porcine model and humans, it is possible that some criteria are not completely compatible and will require further validation in the model. Furthermore, sepsis was induced in young, healthy animals and the translation to elderly intensive care unit (ICU) patients with multiple comorbidities that may affect cardiac calcium handling and mitochondrial function themselves (e.g., heart failure) might be difficult.

The porcine peritonitis-induced sepsis model shows a clear hyperdynamic phenotype and therefore care should be taken to not generalize the results to the hypodynamic phenotypes. It remains, for future studies with more appropriate models, to determine whether or not and to what extent the above described mechanisms contribute to hypodynamic sepsis.

The upstream mechanisms that induce cardiac cellular alterations were not addressed in this study. Extensive research of possible candidates and signaling pathways (e.g., cytokines, oxidative and nitrosative stress, circulating histones) is clearly warranted.

## Author Contributions

JH, LN, and JB conducted the *in vivo* experiments, DJ, JS, LN, MS, and MA participated in cellular and tissue cardiac experiments; MiM and JK conducted the mitochondrial experiments, and MaM and MS conceived of and designed the study and drafted the manuscript. All authors participated in interpretation of the studies, analysis of the data, review of the manuscript, and read and approved the final manuscript.

## Conflict of Interest Statement

The authors declare that the research was conducted in the absence of any commercial or financial relationships that could be construed as a potential conflict of interest.
